# Geochemical Significance of Biomarkers in the Methane Hydrate-Bearing Sediments from the Shenhu Area, the South China Sea

**DOI:** 10.3390/molecules24030456

**Published:** 2019-01-28

**Authors:** Qian-Zhi Zhou, Yan Li, Fang Chen, Shui-Fu Li, Shu-Jun Dong, Feng-Lin Zhang, Xiao-Ming Xu, Jiang-Hai Wang

**Affiliations:** 1 Guangdong Provincial Key Laboratory of Marine Resources and Coastal Engineering, School of Marine Sciences, Sun Yat-Sen University, Guangzhou 510006, China; zhouqzhi@mail2.sysu.edu.cn (Q.-Z.Z.); liyan255@mail.sysu.edu.cn (Y.L.); dongshj3@mail2.sysu.edu.cn (S.-J.D.); zhangfl5@mail2.sysu.edu.cn (F.-L.Z.); 2 Southern Laboratory of Ocean Science and Engineering (Guangdong, Zhuhai), Zhuhai 519000, China; 3 Guangzhou Marine Geological Survey, Guangzhou 510760, China; zhchenfang66@21cn.com; 4 Key Laboratory of Tectonics and Petroleum Resources, China University of Geosciences (Wuhan), Wuhan 430074, China; lishf@cug.edu.cn

**Keywords:** methane hydrates, biomarkers, unresolved complex mixtures (UCMs), microbial activities, the South China Sea

## Abstract

Biomarkers from methane hydrate-bearing sediments can provide vital evidence for microbial activities associated with methanogenesis and their relation to the formation of methane hydrates. However, the former mainly focus on intact polar lipids from these microorganisms, and rarely investigate molecular hydrocarbons such as acyclic isoprenoids and hopanes so far. In this work, the composition of biomarkers in the methane hydrate-bearing sediments in cores SH2B and SH7B from the Shenhu area, the South China Sea (SCS) were identified by gas chromatography-mass spectrometry (GC-MS) and comprehensive two-dimensional gas chromatography/time-of-flight mass spectrometry (GC×GC-TOFMS). The occurrence of unresolved complex mixtures (UCMs) and 25-norhopane indicate that the organic matters in methane hydrate-bearing sediments underwent a high degree of biodegradation. Although specific biomarkers for methanogens were not identified, the UCMs, 25-norhopane, pristane, phytane, and hopanes can still indicate the microbial activities associated with methanogenesis. These molecular signals suggest that diverse microorganisms, particularly methanogens, were quite vigorous in the methane hydrate-bearing sediments. Further, the biomarkers identified in this study can also be steadily detected from deep oil/gas reservoirs. Considering numerous adjacent oil/gas reservoir systems, fault systems, and mud diapers occurred in the SCS, it can be inferred that microbial activities and deep oil/gas reservoirs may have jointly contributed to the formation of methane hydrate deposits in the SCS.

## 1. Introduction

Methane hydrate is a crystalline solid composed of water and methane-dominated hydrocarbon molecules formed under the conditions of low temperature, high pressure, and adequate methane concentrations [1]. It is mainly distributed in the marine sediments and land permafrost [2]. Methane hydrate has many merits, such as wide distribution and abundant resources. It has been estimated that its potential reserves distributed offshore and on land are approximately 1.5 × 10^16^ m^3^; and 97% of the reserves occur offshore [3]. Therefore, methane hydrate is considered as one of the most promising clean energy sources in the 21st century, and has become one potential candidate for the future energy resources compared to oil and coal [4].

Previous studies suggested that microbial methane hydrates were generated by methanogenesis-related microbial communities [5]. Of particular interests are microbial communities and their activities that contribute to the vast formation of hydrated methane in deep-sea sediments [6]; and thus, are potentially critical for the stability, composition, and crystal structures of methane hydrates [7]. Biogeographical distribution and microbial diversity in methane hydrate-bearing sediments have been intensively conducted [6,8,9,10]; and diverse methanogens and methanotrophs were identified in hydrate-related environments [7,11].

The Shenhu area, located in the middle of the northern slope of the South China Sea (SCS), is an important exploration region of methane hydrates. Microbial diversity in the hydrate-containing and -free sediments in the Shenhu area, the SCS, was investigated using 16S rRNA gene phylogenetic analysis, indicating that the presence of hydrates could affect the distribution of microbes [9]. Further, Gong et al. [10] studied the microbial characteristics in the deeply buried sediments in the Taixinan Basin, the SCS, and suggested that the secondary microbial methane generated from the bioconversion of oil or coal was speculated to serve as the enhanced gas flux for the formation of high-saturation methane hydrates.

The Shenhu area is currently the region with the most hydrate drilling samples and geochemical results [12,13]. Previous geochemical studies have demonstrated that the δ^13^C and δD values of methane from hydrates in the Shenhu area range from −71.2‰ to −56.7‰ and −226‰ to −180‰, respectively [14,15], suggesting the predominance of microbial methane in hydrates [13]. However, it is not very persuasive that so many hydrate deposits occurred in the SCS were solely biogenic methane. Further, it is still unclear why methane hydrates are highly saturated in the SCS; and whether the deep oil/gas reservoirs are the gas source for the formation of methane hydrates.

It is well known that the analyses of 16S rRNA and biomarkers can reveal the statuses of methanogens and methanotrophs in deeply buried sediments [16,17]. Notably, the lipid biomarkers from the involved microorganisms are often the key evidence for indicating the nature of their communities in relatively ancient sediments. Up to date, available biomarkers are primarily confined to irregular acyclic isoprenoids and intact polar lipids, including 2,6,10,15,19-pentamethylicosene (PMI), 2,6,11,15-tetramethylhexadecane (crocetane), archaeol, and *sn*-2-hydroxyarchaeol polar lipids, as specific biomarkers for methanogens and methanotrophs [8,18,19], and therefore provide the vital evidence for exploring microbial communities and their related biogeochemical processes. However, the biomarkers, such as pristane, phytane, and hopanes, are rarely investigated up to date. It is well-known that these biomarkers are useful to provide the significant information on the source inputs, thermal maturity, and paleoenvironmental conditions [20]. In addition, these biomarkers may shed light on the oil/source correlation because they can be steadily detected from many oil/gas reservoirs.

In this study, the composition of biomarkers in hydrate-bearing sediments from the Shenhu area, the SCS were analyzed by gas chromatography-mass spectrometry (GC-MS) and comprehensive two-dimensional gas chromatography/time-of-flight mass spectrometry (GC×GC-TOFMS). Our aim is to characterize the microbial activities associated with methanogenesis, and confirm if the oil/gas reservoirs associated hydrocarbons contribute to the substrate for the bioactivities, with emphasis on the method for identifying the biomarkers from deeply buried hydrate-bearing sediments.

## 2. Geological Background

**Figure 1:**
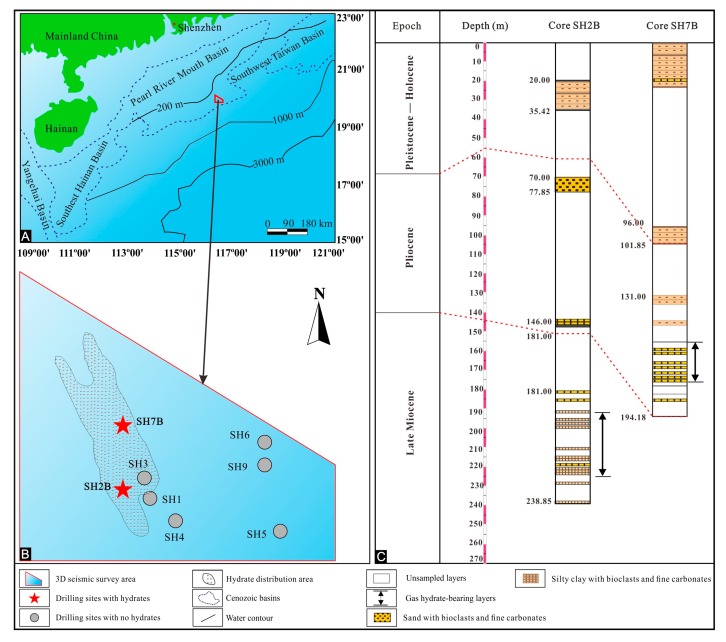
Maps illustrating the locations of sediment cores SH2B and SH7B (**A**,**B**) and stratigraphic columns (**C**) in the Shenhu area, the South China Sea (modified from references [9,24]).

Tectonically, the Shenhu area is located in the Zhu II Depression of the Pearl River Mouth Basin, which has been in a period of tectonic subsidence since the middle Miocene [21,22]. The formation and development of the Pearl River Mouth Basin underwent three main tectonic stages, including Paleocene–early Oligocene fault depression stage, late Oligocene-mid Miocene sag-depression stage, and late Miocene-Quaternary fault blocks-draping and sedimentation stage [12]. The Baiyun Sag with the thick sediment sequence as the depositional center of the Pearl River Mouth Basin is the largest depression during the spreading period of the SCS [23]. The Neogene and Quaternary deep-water sediments with plentiful microfossils in the continental slope were developed in the Shenhu area [24]. The thickness of sedimentary strata in the upper Miocene to the Quaternary mainly ranged from 300 m to 1900 m, with the maximum of 4400 m [23]. The high sedimentation rate resulted in the high contents of organic matters, with the total organic carbon concentrations of 0.46−1.90% in sediments [25]. Two methane hydrate-bearing sediment cores of SH2B and SH7B in this study were collected from the Shenhu area of the northern SCS during scientific expedition conducted by the Geological Survey in 2007, indicating a great prospect for methane hydrate resources in this area [26]. It is notable that methane hydrates mainly occurred in the upper Miocene−lower Pliocene unconsolidated sediments [14]. The hydrate-bearing layers contained plentiful biogenic components, mainly foraminifera, and calcareous ultramicrofossils [24]. Sixteen samples were studied herein, i.e., eight from core SH2B, and eight from core SH7B. The sample information is presented in Table 1 and Figure 1.

## 3. Results and Discussion

### 3.1. Results

**Figure 2:**
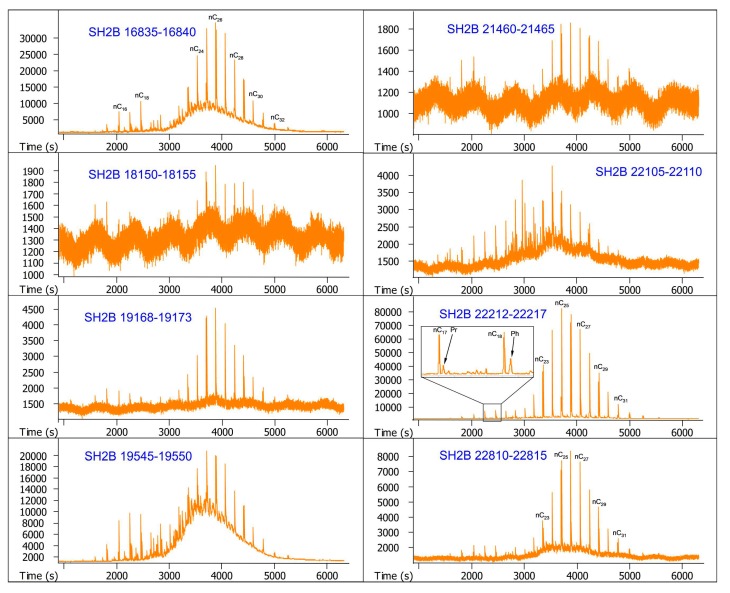
GC-MS chromatograms of the saturated hydrocarbon fractions from sediment core SH2B.The numbers above the main peaks indicate the carbon atom numbers of *n*-alkanes. Pr and Ph indicate pristane and phytane, respectively.

**Figure 3:**
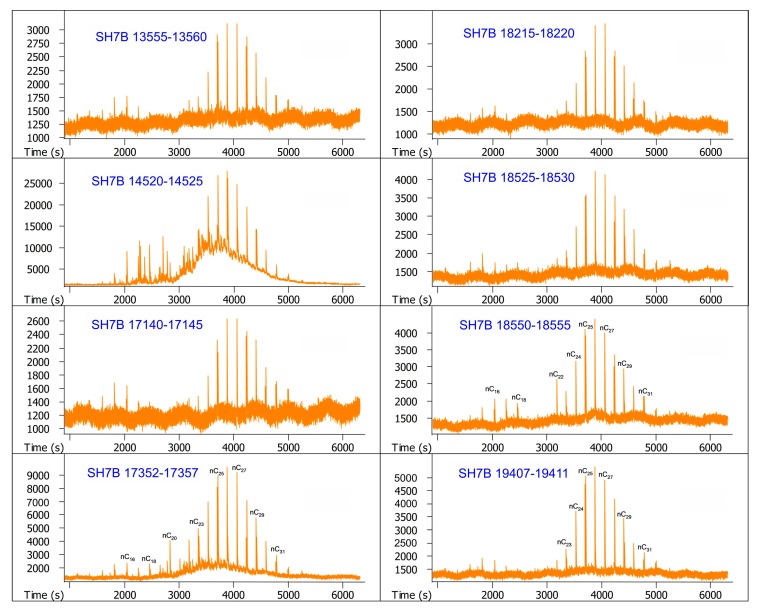
GC-MS chromatograms of the saturated hydrocarbon fractions from sediment core SH7B. The numbers above the main peaks indicate the carbon atom numbers of *n*-alkanes. Pr and Ph indicate pristane and phytane, respectively.

The GC-MS chromatograms of samples are illustrated in Figure 2 and Figure 3. It can be seen from the two figures that very few hydrocarbons are identified from these samples, but unresolved complex mixtures (UCMs) occur as a big hump associated with the long-chain *n*-alkanes in most of them. As a whole, *n*-alkanes for every sample are primarily concentrated in a relatively narrow range of *n*C_22_–*n*C_30_, and characterized by the unimodal distribution with a maximum abundance at *n*C_26_. It is noteworthy that pristane and phytane are identified with the feature that phytane is always more abundant than pristane, but terpanes and steranes are undetectable.

**Figure 4:**
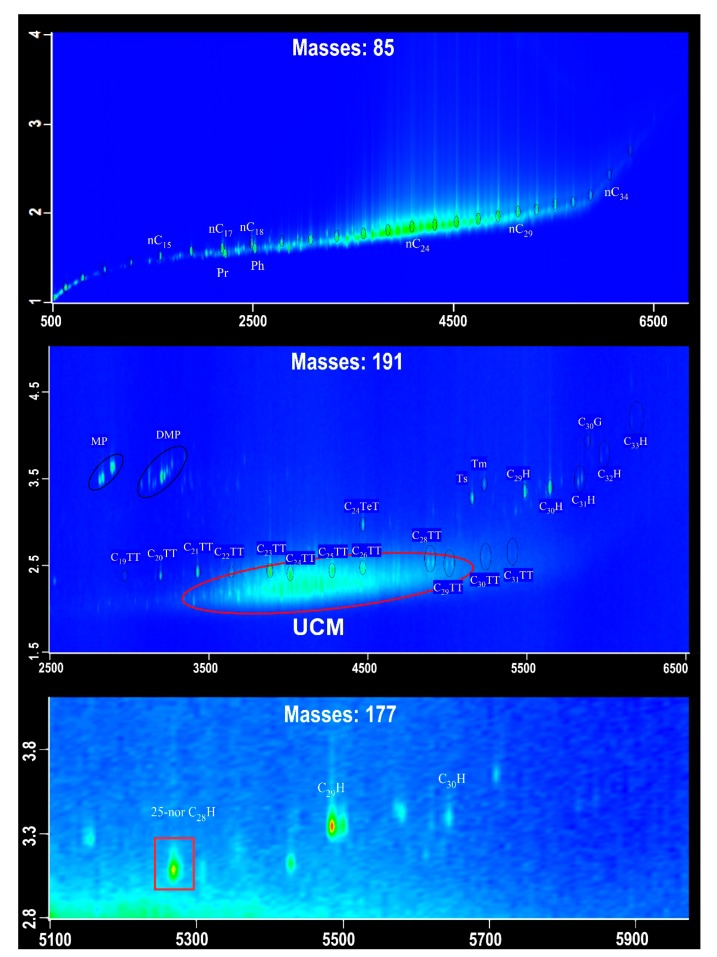
Representative two-dimensional chromatograms of fragment ions *m*/*z* 85, 177, and 191. UCM, unresolved complex mixtures; MP, methylphenanthrene; DP, dimethylphenanthrene; TT, tricyclic terpane; H, hopane; C_30_G, gammacerane. The numbers above the main peak indicate the carbon atom numbers of *n*-alkanes (*m*/*z* 85), tricyclic terpanes, and hopanes (*m*/*z* 191 and 177). Pr and Ph indicate pristane and phytane, respectively.

Because of the low contents of soluble organic matters in sediments, only samples 4 and 10 could be further analyzed by GC×GC-TOFMS. As we know, GC×GC-TOFMS has a strong capacity to identify numerous biomarkers in comparison with conventional GC-MS. These biomarkers mainly include *n*-alkanes, pristane, phytane, tricyclic terpanes, hopanes, and 25-norhopane (Figure 4). It is notable that steranes were still undetectable in this study. Methylphenanthrene (MP) and dimethylphenanthrene (DMP) were identified in *m*/*z* 191 mass chromatograms, and might come from the alumina/silica gel columns at the time of separation. The composition of *n*-alkanes detected by GC×GC-TOFMS is generally consistent with that by GC-MS. Tricyclic terpanes (TT) are primarily distributed in the range of C_19_TT–C_31_TT; and C_24_ tetracyclic terpane (C_24_TeT) is the only one detected from tetracyclic terpanes. Hopanes mainly consist of C_27_ to C_33_ 17α, 21β hopanes, and 17α, 21β C_30_ hopane is the major compound. The Ts/(Ts + Tm) ratios for samples 4 and 10 are 0.66 and 0.77, respectively. Gammacerane and 25-norhopane were also detected but with the low abundances. Overall, GC×GC-TOFMS analysis has a high resolution for clearly identifying the biomarkers, which cannot be separated by conventional GC-MS.

### 3.2. Discussion

Methanogenesis as the final process in the fermentation of organic matters is performed by the combination of methanogens and other anaerobic microbes [27]. Generally, there are two primary pathways of methanogenesis, namely CO_2_ reduction and acetate fermentation [28]. There are also other pathways of methanogenesis, i.e., methane to be produced from methanol [29]. It has been proposed that microbial methane in the Shenhu area was generated via CO_2_ reduction on the basis of its δ^13^C and δD values [13]. Because most of methanogens cannot directly utilize complex organic matters, methanogenesis depends on the synergistic degradation of various microbial communities [30].

The biomarker evidence from hydrate-bearing sediments may insight into biogeochemical processes associated with methanogenesis [31]. In this study, UCMs occurred in most of the samples, and in particular 25-norhopane was further detected by GC×GC-TOFMS. The technique of GC×GC-TOFMS greatly improves the separation capacity of the chromatographic system [32,33,34,35,36,37,38,39]; and thus, enables us to analyze minor components in the extracts of sediments/rocks. Generally, 25-norhopane and UCMs have been commonly ascribed to biodegradation and/or microbial activities [20,38]. This means that the organic matters in the Shenhu area, the SCS underwent a high-degree biodegradation. Although the composition of UCMs was not measured by GC×GC-TOFMS in this study, it has been suggested that UCMs contain even up to 250,000 compounds [38,40]. These compounds may represent the products of anaerobic microbial degradation of complex organic matters. It is known that abundant degradation products, which can be used by methanogens, are of significant importance in the production of methane [41]. This means that the high input of low-molecular-weight organic matters derived from microbial activities leads to strong methanogenesis populations in sediments. In addition, the higher abundances of phytane indicate an anoxic depositional environment [20], which is consistent with the widely-distributed pyrite in sediments [42]. The occurrence of anoxic conditions is beneficial for the survival of anaerobic microbial activities.

It is of significant importance to have abundant microbes in the methane hydrate area, because methanogenesis should be performed by the combination of methanogens and other anaerobic microbes. Commonly, these microbes will leave their specific biomarkers, which may be preserved in hydrate-bearing sediments. Therefore, we can obtain the biological information associated with methanogenesis from these biomarkers. Unfortunately, specific biomarkers for methanogens, such as 2,6,10,15,19-pentamethylicosenes (PMEs), C_19_–C_33_ acyclic isoprenoids, and squalene [43,44], were not identified from aliphatic hydrocarbons in this study. This may be due to their low abundances, and thus these microbial signals can be easily obscured by UCMs. In fact, the intact polar lipids have revealed the occurrence of methanogens in the deeply buried sediments [8,18]. In addition, 16S rRNA sequencing of hydrate-bearing sediments at the similar depths in the Taixinan Basin, the SCS also showed the coexistence of syntrophic bacteria and methanogens [10].

Although no specific biomarkers for methanogens were detected, other biomarkers associated with methanogenesis, including acyclic isoprenoids (pristane and phytane) and hopanes, were still identified in this study. Generally, pristane and phytane are originated from a similar chlorophyll source, i.e., photosynthetic products of algae or cyanobacteria [45]. However, pristane can be also derived from methanogens; while halophilic bacteria can generate phytane [20]. In addition, regular C_19_–C_33_ acyclic isoprenoids are indicators for strictly anaerobic methanogens [43]. Most of these components contain a pristane unit linked head-to-head with other isoprenoid units. Therefore, pristane and phytane probably linked with the activities of methanogens.

Hopanes originate from the cytomembrane of prokaryotic bacteria, mainly including cyanobacteria, heterotrophic bacteria and chemoautotrophic bacteria [46]. A series of tricyclic terpanes may be the biomarkers derived from higher terrestrial plants or bacteria [47]. It is well known that the ratio of hopanes to steranes (Hop/Ste) can reflect the relative contribution between bacteria and algae [20]. Unfortunately, we did not detect steranes by both GC-MS and GC×GC-TOFMS, probably implying a very little input of eukaryotes. In spite of the occurrence of 25-norhopane and UCMs that denote biodegradation, the largely unchanged distribution of *n*-alkanes in those samples suggests that the absence of steranes did not result from biodegradation because *n*-alkanes were preferentially biodegraded, and accordingly depleted relative to steranes [20]. Thus, the detection of abundant biomarkers indicates that the microbes associated with methanogens were quite numerous in hydrate-bearing sediments in the Shenhu area, the SCS. This may be one of the most important reasons for the formation of hydrate deposits in this area.

However, the indigeneity of biomarkers is crucial for geochemical interpretation. In fact, the biomarkers determined in this study can be steadily detected from oil/gas reservoirs [48,49,50]. Moreover, the existing studies have demonstrated that there are numerous nearby oil/gas reservoir systems, such as in the Qiongdongnan Basin, the Pearl River Mouth Basin, and the Taixinan Basin [23,51,52,53]. The Ts/(Ts + Tm) ratios for samples 4 and 10 also reveal a high thermal maturity [20]. These observations imply that the oil/gas reservoirs may partially contribute to the supply of hydrocarbons in this case, and accordingly provide the plentiful carbon source and hydrogen for methanogens [41]. This is consistent with the previous microbial evidence [10]. In addition, the existence of fault systems and mud diapers in the SCS indicated the probable pathways for oil/gas migration [54,55]. The above evidence comprehensively indicates that the microbial activities together with deep oil/gas reservoirs may contribute to the formation of hydrates in the SCS.

## 4. Methods

### 4.1. Extraction and Separation

The solvents for extraction and separation were of HPLC grade. Glassware was combusted in an oven at 400 °C for 4 h before our experiments. Filter paper, alumina and silica gel were extracted by dichloromethane (DCM) to remove any possible contaminant.

The samples were crushed to less than 100 meshes after freeze-drying, and about 40 g of the crushed samples were Soxhlet extracted for 72 h using a solvent mixture of DCM and methanol (3:1, *v/v*), with the activated copper added to remove any elemental sulfur that was extracted. After removal of the solvents and asphaltenes, the resultant maltene fraction was separated into aliphatic hydrocarbons, aromatic hydrocarbons, and polar compounds using alumina/silica gel column chromatography. The saturated fractions from all samples were analyzed by GC-MS. Samples 4 and 10 were further analyzed using GC×GC-TOFMS.

### 4.2. GC-MS and GC×GC-TOFMS Analysis

GC-MS analysis was performed using an Agilent 6890 gas chromatographic interfaced with an Agilent 5973 mass spectrometer (Agilent Technologies, Palo Alto, CA, USA). The GC was equipped with a DB-5 MS fused silica capillary column (J&W Scientific, Agilent, USA; 60 m × 0.25 mm i.d. × 0.25 μm). Helium was supplied as the carrier gas at a flow rate of 1.0 mL/min. The injector and detector temperatures were set at 290 °C and 300 °C, respectively. After concentrated, 2 μL of each sample was injected in the splitless mode. The GC oven temperature was initially set at 80 °C (held for 5 min), and was then programmed at 3 °C/min to 290 °C (held for 20 min).

A LECO Pegasus 4D GC×GC-TOFMS instrument (Leco Corp., St Joseph, MI, USA) that consisted of an Agilent 7890A GC (Agilent Technologies, Palo Alto, CA, USA) equipped with a secondary-liquid nitrogen-cooled pulse-jet modulator was used. The GC column system (non-polar/polar, NP/P) composed of an HP–5MS (60 m × 0.25 mm i.d. × 0.25 μm) coupled with a DB-17HT (3 m × 0.1 mm i.d. × 0.1 μm).

The GC injector temperature was set at 310 °C (>2 μL injection) with a splitless mode; and helium was used as the carrier gas at a constant flow of 1.5 mL/min. A primary oven temperature program was adopted in this analysis, i.e., initially held at 40 °C for 2 min, and increased to 310 °C at a rate of 2 °C/min with a final hold of 16 min. The second oven temperature programmed from 130 °C (2 min) to 310 °C at 3 °C/min, and held isothermally for 30 min. The modulator temperature was 30 °C higher than the primary, and the modulation period was 8 s with 2 s hot-pulse time. The temperature of the transfer line was 280 °C. The TOFMS analysis was performed under the following conditions: electron energy of 70 eV; a −1475 V detector voltage; a 240 °C ion source temperature; an acquisition rate of 100 spectra per second; and a scan range of 40–520 amu. The acquisition delay time was 600 s. Chroma TOF software ver. 4.24 was used to process the data; and the compound assignment was performed by examination and comparison with literature mass spectra, retention time, and elution order, as well as using the NIST05 library of mass spectra [20,36,37,38].

## 5. Conclusions

GC×GC-TOFMS analysis can clearly identify the biomarkers that cannot be separated by conventional GC-MS. UCMs, 25-norhopane, pristane, phytane, and hopanes have been identified from deeply buried hydrate-bearing sediments in the Shenhu area, in the SCS. The occurrence of these biomarkers suggests that the organic matters in sediments underwent a high degree of biodegradation; and, that there were microbial activities vigorously involved in methanogenesis. In fact, the identified biomarkers in this study can also be steadily detected from oil/gas reservoirs. Due to the occurrence of many nearby oil/gas reservoir systems, fault systems, and mud diapers in the SCS, we consider that the microbial activities together with deep oil/gas reservoirs may contribute to the formation of hydrate deposits in the SCS.

## Figures and Tables

**Table 1:** Sample information.

Sample Number	Core Number	Depth Interval (cm)	Lithology	Layers
1	SH2B	16,835–16,840	Silty clay	Overlying layer
2	SH2B	18,150–18,155	Silty clay	Overlying layer
3	SH2B	19,168–19,173	Silty clay	Methane hydrate-bearing layer
4	SH2B	19,545–19,550	Silty clay	Methane hydrate-bearing layer
5	SH2B	21,460–21,465	Silty clay	Methane hydrate-bearing layer
6	SH2B	22,105–22,110	Silty clay	Methane hydrate-bearing layer
7	SH2B	22,212–22,217	Silty clay	Methane hydrate-bearing layer
8	SH2B	22,810–22,815	Silty clay	Underlying layer
9	SH7B	13,555–13,560	Sand	Overlying layer
10	SH7B	14,520–14,525	Sand	Overlying layer
11	SH7B	17,140–17,145	Silty clay	Methane hydrate-bearing layer
12	SH7B	17,352–17,357	Silty clay	Methane hydrate-bearing layer
13	SH7B	18,215–18,220	Silty clay	Underlying layer
14	SH7B	18,525–18,530	Silty clay	Underlying layer
15	SH7B	18,550–18,555	Silty clay	Underlying layer
16	SH7B	19,407–19,411	Silty clay	Underlying layer
